# Geospatial Analysis of Organ Transplant Referral Regions

**DOI:** 10.1001/jamanetworkopen.2022.31863

**Published:** 2022-09-15

**Authors:** Tyler Schappe, Sarah Peskoe, Nrupen Bhavsar, L. Ebony Boulware, Jane Pendergast, Lisa M. McElroy

**Affiliations:** 1 Duke University, School of Medicine, Durham, North Carolina

## Abstract

**Importance:**

System and center-level interventions to improve health equity in organ transplantation benefit from robust characterization of the referral population served by each transplant center. Transplant referral regions (TRRs) define geographic catchment areas for transplant centers in the US, but accurately characterizing the demographics of populations within TRRs using US Census data poses a challenge.

**Objective:**

To compare 2 methods of linking US Census data with TRRs—a geospatial intersection method and a zip code cross-reference method.

**Design, Setting, and Participants:**

This cohort study compared spatial congruence of spatial intersection and zip code cross-reference methods of characterizing TRRs at the census block level. Data included adults aged 18 years and older on the waiting list for kidney transplant from 2008 through 2018.

**Exposures:**

End-stage kidney disease.

**Main Outcomes and Measures:**

Multiple assignments, where a census tract or block group crossed the boundary between 2 hospital referral regions and was assigned to multiple different TRRs; misassigned area, the portion of census tracts or block groups assigned to a TRR using either method but fall outside of the TRR boundary.

**Results:**

In total, 102 TRRs were defined for 238 transplant centers. The zip code cross-reference method resulted in 4627 multiple-assigned census block groups (representing 18% of US land area assigned to TRRs), while the spatial intersection method eliminated this problem. Furthermore, the spatial method resulted in a mean and median reduction in misassigned area of 65% and 83% across all TRRs, respectively, compared with the zip code cross-reference method.

**Conclusions and Relevance:**

In this study, characterizing populations within TRRs with census block groups provided high spatial resolution, complete coverage of the country, and balanced population counts. A spatial intersection approach avoided errors due to duplicative and incorrect assignments, and allowed more detailed and accurate characterization of the sociodemographics of populations within TRRs; this approach can enrich transplant center knowledge of local referral populations, assist researchers in understanding how social determinants of health may factor into access to transplant, and inform interventions to improve heath equity.

## Key Points

**Question:**

How can transplant referral regions be optimally characterized using demographic data from the US Census?

**Findings:**

This cohort study found that use of a geospatial method to model transplant referral regions avoided duplicative assignments and resulted in a significantly reduced incorrect assignment area compared with a zip code cross-reference method.

**Meaning:**

These results suggest that accuracy in linking transplant referral regions to demographic Census data can be improved to allow more detailed and specific characterization of the social determinants of health.

## Teaser

This cohort study of US adults on kidney transplantation waiting lists compares 2 models for characterizing transplant referral regions using US Census data.

## Introduction

Inequities in access to organ transplant are longstanding, having been described for over 20 years.^1^ Women, racial and ethnic minority populations, and patients with public insurance, low educational level, and low income all have lower rates of listing for organ transplant, higher rates of waitlist mortality, and lower rates of transplant from living donors.^2,3,4,5,6,7,8,9,10,11,12,13,14,15,16,17,18,19,20,21,22,23,24,25,26,27^ Transplantation is a highly complex, often regionalized system of care that requires completion of a multi-step conditional selection process. Inequities in access to transplantation likely result from a combination of the complexity of the transplant selection process and individual social conditions (eg, health literacy, employment, access to transportation) that burden patients as they navigate the process.

Transplant centers have a unique and important role in facilitating equitable access to transplant by optimizing the systems and the processes by which they evaluate, select, and maintain communication with transplant patients. Numerous potential social challenges may impede access to transplantation, and developing and implementing center-level interventions requires a detailed understanding of the most significant and prevalent social barriers faced by the referral populations of each center. Transplant referral regions (TRR) are geographic areas that represent the population most commonly referred to each transplant center in the US.^28^ TRRs were originally derived in 2014 for patients with end-stage kidney disease to assess differences in demographically adjusted rates of waitlisting and transplantation and to assess outcomes associated with variations in access to transplant care on overall end-stage kidney disease–related mortality.^29^ The method expanded in 2020, deriving TRRs for liver, pancreas, heart, and lung transplant centers.^30^

Current TRRs are derived using geographic US Postal Service zip codes, an areal unit that exists for the sole purpose of guiding mail delivery. Although common, the use of zip codes for epidemiologic purposes or to determine health care delivery can lead to misclassification of structural social determinants of health and overestimation or underestimation of community deprivation.^31,32,33,34^

The goal of this retrospective cohort study was to expand on the methodology of generating TRRs from zip codes to the census tract and block levels, and to compare a zip–hospital referral region (HRR) cross-reference method (published by Dartmouth Health Atlas) with direct geospatial intersection in order to improve the ability to link social determinants of health data to TRRs. Our hypothesis was that the geospatial intersection method of linking TRRs to American Community Survey data would result in reduced error and allow improved characterization of the communities associated with each center.

## Methods

### Data Sources

We obtained transplant center spatial data from the Health Resources Services Administration (HRSA), which included a list of currently active transplant centers with global positioning system coordinates.^35^ Care utilization spatial data included a zip-to-HRR cross-reference and HRR polygons, census tract and census block group polygon features, and a zip code tabulation area to census tract cross-reference.^36,37,38,39,40^

The United States Renal Data System (USRDS) provided patient waitlist data, along with cross-reference files for linking internal facility identifiers to 4-letter over the counter (OTC) codes as listed in the HRSA website and for linking multiple facility identifiers that refer to the same facility over time.^41^ This study was approved by the Duke University institutional review board.

### Study Period and Cohort Definitions

The study period was January 1, 2008, to December 31, 2018. Waitlist entries that met the following criteria were included: (1) occurred at a USRDS facility that has performed at least 1 transplant, (2) at least 1 day of the time on the waitlist period occurred during the study period, (3) waitlist start date was not missing and did not occur after the recorded waitlist end date, and (4) portion of waitlist during which patient was aged 18 years or older. We assumed that waitlist end dates that were missing represented patients who were active on the waitlist as of the date of creation of the data set and we administratively censored waitlist entries on the last day of the study period.

### Transplant Referral Region Derivation

We derived TRRs based on the method described by Ross et al.^30^ We combined transplant centers that were located within 10 straight-line miles of each other since their highly overlapping patient populations make distinguishing separate catchment areas difficult. We also combined centers that did not meet this criteria but were linked by a third center that was within 10 miles of both were; this only occurred in New York and Chicago metropolitan areas. We assigned each waitlist entry that met the inclusion criteria to a corresponding HRR based on the patient home zip code using the zip to HRR cross-reference. We allocated waitlist entries for patients whose home zip is associated with multiple HRRs in the cross-reference to all relevant HRRs.

We counted waitlist entries in each HRR and assigned the HRR to the transplant center (or combined group of centers) at which the majority of patients in that HRR were waitlisted during the study period. During this process, a patient with multiple nonoverlapping waitlist entries at a single center only counted once for that center, but waitlist entries at multiple centers for the same patient counted for all their respective centers. Facilities identified as having multiple facility identification numbers over time were present in the USRDS-OTC cross-reference; therefore, we combined these redundant identifications into their respective OTC codes upon merging. We assigned a single OTC code to 2 centers and removed the duplicate center. In total, 38 OTC codes were dropped since they were not present in the HRSA listing and likely represent centers that were not active as of the creation of the HRSA data set. We spatially combined (ie, dissolved) HRRs assigned to the same transplant center to form the center’s TRR.

### Aggregating Census Tract- and Census Block Group-Based Data

We compared 2 methods for linking and aggregating demographic data observed at the census tract or census block group spatial scales to the derived TRRs: 5-digit zip code cross-reference and direct geospatial intersection. For the zip code cross-reference method, we first joined the zip code to census tract cross-reference file with the zip code to HRR cross-reference file by zip code to obtain an HRR for each census tract. We then joined the resulting data set with the derived TRR data set by HRR to assign a TRR to each census tract. To obtain an analogous data set for census block groups, we joined the census tract to TRR cross-reference data set with a data set of census block groups by the unique GEOID of parent census tracts; in this process, all census blocks groups within the parent census tract were assigned to the TRR of the parent.

In the spatial intersection method, we first geoprocessed census tracts and block group polygons to exclude coastlines and other unpopulated areas not assigned to HRR polygons. To do this, we spatially combined (dissolved) all HRR polygons to create a single large HRR polygon, and we then found the intersections of all census tracts and block with this large HRR polygon. We then identified all pairwise spatial intersections between each TRR and either census tract or census block groups. Finally, we assigned each census tract or block group to the TRR with the largest area of intersection.

### Statistical Analysis

We used 2 metrics to assess the accuracy of each of the 2 methods—quantifying multiplets and quantifying area misassigned*.* In cases where a census tract or block group crosses the boundary between 2 HRRs that were assigned to different TRRs, this results in the demographic data associated with the census unit being aggregated to multiple TRRs, effectively including the same population and associated demographics multiple times erroneously. In order to quantify how often this type of overrepresentation occurred, we counted the number of census tracts and census block groups that were associated with more than a single TRR.

For quantifying areas that were misassigned, we defined misassigned areas as consisting of 2 components: (1) portions of census tracts or block groups assigned to a TRR but located outside of the boundaries of that TRR and (2) portions of TRRs not assigned any demographic data. This type of error is distinct from multiplets because the same data are not necessarily used multiple times. However, it does still represent error in that population and associated demographic characteristics located in one TRR are being ascribed to another. To calculate each of these components, we found the symmetric difference in area between each TRR polygon and the census units assigned to that polygon. We then summed the area resulting from each of these components to find the total area misassigned for each TRR. We calculated the percentage of area misassigned in each TRR as the total misassigned area divided by the total area of the TRR. We used paired Wilcoxon signed rank tests to assess differences in median area and percentage of TRR area misassigned between methods.

We performed all analyses, including geoprocessing, and we generated all figures and tables using R version 4.0.4 (R Project For Statistical Computing).^40,42,43,44,45,46,47,48,49,50,51,52,53,54,55^ We utilized the s2 spherical geometry library available in the sf R package for geoprocessing, including finding intersections and calculating geographic area, using geographic rather than projected coordinates, which enabled a large reduction in computation time. We performed a validation for a subset of TRRs in which we performed all analyses using both s2 spherical geometry and standard planar geometry after projecting all polygons to North America Albers Equal Area Conic coordinates and found no meaningful difference between the 2 approaches.

## Results

**Figure 1.**
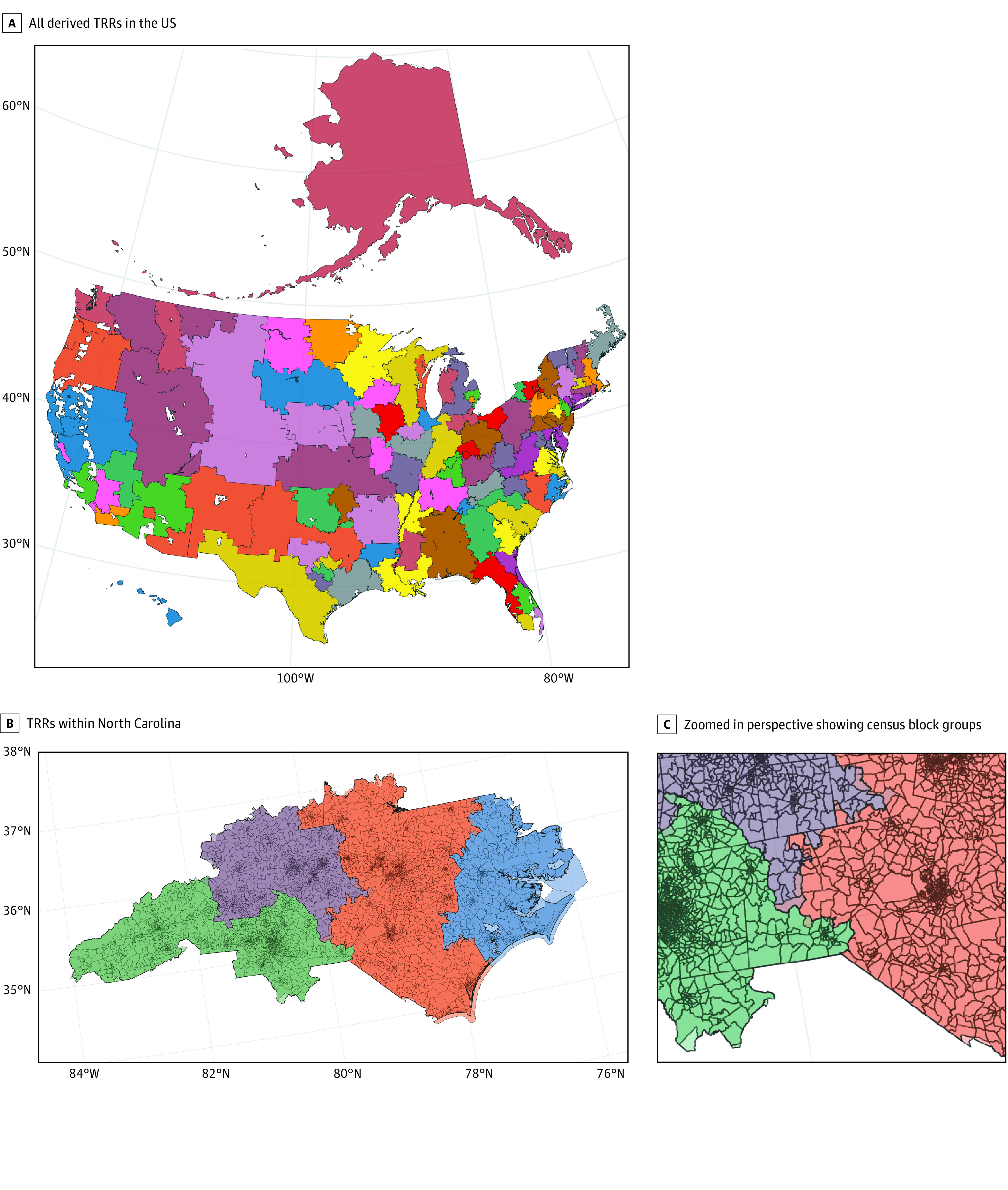
Maps Illustrating Nesting of Census Block Groups Within Derived Transplant Referral Regions (TRRs) Discolored areas near TRR boundaries indicate error from spatial method.

Between 2008 and 2018, a total of 359 794 adults were active on the kidney transplant waitlist at 261 transplant centers in the US, represented by 434 409 waitlist entries. In total, we defined 102 TRRs for 238 transplant centers by assigning waitlisted patients to centers, then patient home zip codes into HRRs and TRR polygons (Figure 1). A total of 4459 waitlist entries were dropped because the patient zip code was missing from the zip-HRR cross-reference file (2984 [66.9%] of these were waitlist entries from Puerto Rico, where HRRs are not defined). In addition, 11 252 waitlist entries at 38 transplant centers were dropped because their OTC codes were not available in the current HRSA listing of active centers.

### Zip Code Cross-Reference Method

Across the 102 TRRs, a median of 528 (range, 78-3412) census tracts and 1584.5 (range, 242-10 930) census block groups were assigned to TRRs based on their zip codes. The zip code cross-reference method resulted in 7657 census block groups (3.5%) and 2449 census tracts (3.4%) being erroneously assigned to multiple TRRs, representing 18% of the total area of the US covered by a TRR (Table 1). Among all 102 derived TRRs, this method resulted in absolute misassigned area ranging from 120 to 121 000 km^2^, with a median of 8330 km^2^. This method also gave a percentage of area misassigned relative to total TRR area ranging from 1.4% to 87.9%, with a median of 20.4% at both census tract and block group spatial scales (Table 2).

**Table 1.** Census Tracts and Block Groups Assigned to Multiple TRRs by Absolute Area and Percentage Area

Method	Spatial unit	No. double-assigned	Area double-assigned, km^2^ (%)
Spatial	Block group	0	0
	Census tract	0	0
Zip	Block group	7657	1 641 994 (18.0)
	Census tract	2449	1 641 999 (18.0)

Abbreviation: TRRs, transplant referral regions.

**Table 2.** Misassigned TRR Area

Method	Block group			Census tract		
	Spatial (N = 102)	Zip (N = 102)	*P* value	Spatial (N = 102)	Zip (N = 102)	*P* value
TRR misassigned, %						
Mean (SD)	4.1 (3.1)	22.9 (14.9)	NA	6.5 (4.8)	22.9 (14.9)	NA
Median (range)	3.5 (0.2-23.9)	20.4 (1.4-87.9)	<.001	5.3 (0.6-31.9)	20.4 (1.4-87.9)	<.001
Area misassigned, km^2^						
Mean (SD)	2860 (4510)	16 600 (24 000)	NA	4330 (6750)	16 600 (24 000)	NA
Median (range)	1150 (36-27 200)	8330 (120-121 000)	<.001	1970 (36-40 100)	8330 (120-121 000)	<.001

Abbreviation: TRRs, transplant referral regions.

### Spatial Intersection Method

Across the 102 TRRs, a median of 499.5 (range, 74-3403) census tracts and 1497.5 (range, 229-10861) census block groups were assigned to the TRR with the largest area of overlap. The spatial intersection method resulted in exactly zero census tracts or census block groups assigned to multiple TRRs (Table 1). Among all 102 derived TRRs, this method resulted in a median absolute misassigned area of 1970 km^2^ at the census tract scale and 1150 km^2^ at the census block group scale. Expressed as the percentage of misassigned area relative to total TRR area among all TRRs, this method resulted in a median of 5.3% (range, 0.6%-31.9%) at the census tract scale and 3.5% (range, 0.2%-23.9%) at the census block group scale (Table 2).

### Comparison of Methods

**Figure 2.**
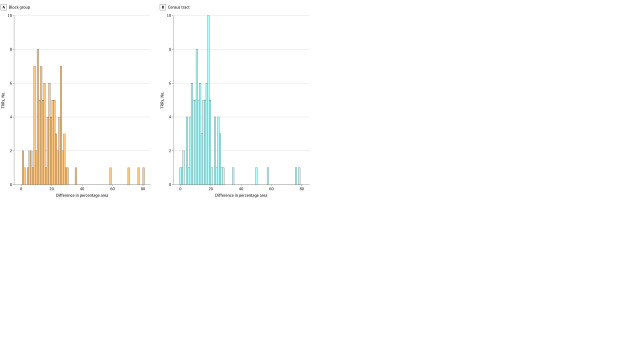
Histograms of Difference in Area Misassigned as a Percentage of Total Transplant Referral Region (TRR) Area Between Zip Code Crosswalk Method and Spatial Method by Spatial Unit at Both Census Tract and Census Block Group Spatial Scales The direction of the difference is the zip method minus the spatial method, so that positive values indicate larger percentage of area misassigned for the zip method.

The spatial method eliminated the issue of census tracts or block groups being assigned to multiple TRRs, while the zip code crosswalk method resulted in 7657 census block groups and 2449 census tracts (18.0% of total TRR area) being multiply assigned. In every TRR, the spatial method resulted in equal or less area misassigned compared with the zip crosswalk method (Figure 2). Compared with the zip crosswalk method, the spatial method resulted in a reduction in the mean and median percentage area misassigned, respectively, of 16.4% (22.9% vs 6.5%) and 15.1% (20.4% vs 5.3%) for census tracts and 18.8% (22.9% vs 4.1%) and 16.9% (20.4% vs 3.5%) for census block groups (Table 2). The spatial method resulted in significantly less area misassigned than did the zip code method, both at the census tract and census block group scales (*P* < .001 in Wilcoxon signed-rank tests).

## Discussion

In complex, regionalized care systems, health system or center–driven efforts may be uniquely suited to achieve sustained improvements in health equity by redesigning networks and processes of care to focus on mitigating patient-level barriers while remaining resilient to institutional and interpersonal bias. This requires a detailed understanding of the unique needs of the general population that may reside in a wide geographic area. With that in mind, the goal of this study was to explore optimal ways of characterizing general populations within TRRs using data from the American Community Survey. In doing so we sought to facilitate improved characterization of social determinants of health, as well as to compare 2 methods of aggregating census tract and census block group data with TRRs: spatial intersection and zip crosswalk. Our work revealed that for every TRR, the spatial intersection method resulted in equal or less area misassigned compared with the zip crosswalk method. Compared with the zip crosswalk method, the spatial intersection method resulted in a reduction in the mean and median percentage area misassigned, respectively, and can be performed relatively easily using established R packages.

Health inequities are inextricably linked to structural racism, whereby historically marginalized groups are limited in their opportunities, resources, and well-being due to macro-level conditions.^56^ Social determinants of health are known drivers of inequities in access to complex care, but understanding how social determinants of health factor in the ability of patients to successfully access the transplant waitlist and undergo transplantation is limited by lack of actionable data.^17,19,57,58,59,60,61,62,63,64,65,66,67^ National data registries of transplant patients include few social determinants of health and have limited reliability, and patients with chronic organ disease are poorly represented in most public health databases.^68^ Prior work has used zip code as a proxy for community-based social determinants of health. This has several disadvantages. Zip codes represent a larger population size and are associated with potential greater heterogeneity, particularly in densely populated urban communities.^32^ Zip codes are also administrative units established for mail delivery rather than to characterize populations, and may change over time based on local real estate and urban development.^33^ A recent investigation of misalignment between zip codes and municipal boundaries in Michigan found that only 14% of Michigan's land area matches zip code and municipality name, which correlated with 49% of the population being misrepresented in some way by their zip code.^31^

### Limitations

This study had several limitations. Our analysis involved linkage of USRDS, HRSA, and census data and as such was subject to the limitations inherent in large registries. In particular, waitlist entries for patients whose home zip was associated with multiple HRRs in the cross-reference would be allocated to all relevant HRRs; this is an unavoidable consequence of having low spatial resolution for patient home locations. While characterizing misassignment with respect to population as opposed to geographic area would likely be more informative, given the methodological challenges this poses, further investigation would be needed. For example, because census block groups are the smallest spatial scale available from the American Community Survey, it was not possible with the available data to obtain the population for a portion of a census block group. At the census tract scale, constituent census block groups could potentially be utilized to quantify population misassigned, but the disjoint borders of TRRs and block groups would cause additional complications and error. As such, we reported our magnitude of misassignment associated with each method using geographic area.

An alternative approach to assigning entire source polygons to the single target TRR with maximum area overlap is areal interpolation. In this approach, census tracts or block groups are divided based on the TRR boundaries and the component polygons are assigned to overlapping TRRs; weighted averages of demographic data are then assigned to the components using weighting based on area or population. Areal interpolation would result in no misassignment error since census tracts or block groups would be subdivided, but relies on the strong assumption of homogeneity of data within polygons (in the case of area-weighted interpolation)^69^ or additional population data at a finer spatial scale than the component polygons (in the case of population-weighted areal interpolation).^51^ TRRs depend on patient-level waitlist data, and their derivation is vulnerable to ecologic and individualistic fallacy.^70^

Transplantation remains a rapidly evolving field and the results of this study were a static representation of a dynamic process where ultimately patients can travel anywhere in the country to be listed. However, a small minority of patients were listed in geographically disparate and remote centers relative to their residence. The authors also maintain that transplant centers should be active participants in the communities in which they reside and focus primary outreach efforts on patients with end-stage organ disease. We chose to focus this study on a detailed description of our rationale and methods for how to associate demographic data with TRRs using spatial methods. While we did compare methods with respect to geographic error, we did not include a comparison of results by sociodemographic characteristics by method in this analysis. However, at the time of publication our group was beginning to examine variation in sociodemographic composition of TRRs, and we leave this as the subject of a future study. Finally, we have linked demographic data to TRRs at the census block level to facilitate more detailed characterization of neighborhoods and communities surrounding transplant centers. We hope this will improve understanding of structural and institutional racisms, but recognize those efforts will need to occur in parallel to, rather than instead of, those that address interpersonal racism.

## Conclusions

Organ transplantation is a multidisciplinary, highly complex therapy primarily available at regional centers, and a well-suited model for understanding how referral population characteristics vary around the US is needed. Linking TRRs to demographic data at the census tract or census block group spatial scale using a spatial intersection method avoids errors due to duplicate and incorrect assignments, and greatly reduces the amount of misassignment error compared with a zip code crosswalk method. This spatial method provides increased spatial resolution, complete coverage of the country, and more balanced population counts. It also enables more detailed and accurate characterization of the sociodemographic characteristics of referral populations. This approach can enrich transplant center knowledge of local referral populations, assist researchers in understanding the influence of social determinants of health on access to transplant, and inform interventions to improve heath equity.
